# Dextran Nanocapsules with *ω*-3 in Their Nucleus: An Innovative Nanosystem for Imiquimod Transdermal Delivery

**DOI:** 10.3390/pharmaceutics14112445

**Published:** 2022-11-11

**Authors:** Gisela F. Carreño, María Javiera Álvarez-Figueroa, José Vicente González-Aramundiz

**Affiliations:** 1 Departamento de Farmacia, Escuela de Química y Farmacia, Facultad de Química y de Farmacia, Pontificia Universidad Católica de Chile, Santiago 7820436, Chile; 2 Centro de Investigación en Nanotecnología y Materiales Avanzados “CIEN-UC”, Pontificia Universidad Católica de Chile, Santiago 7820436, Chile

**Keywords:** dextran nanocapsules, nanocapsules, dextran sulfate, imiquimod, transdermal delivery, skin, chemical enhancer of penetration

## Abstract

Transdermal administration of molecules across the skin has gained interest because it can be considered a non-invasive route compared with traditional ones. However, going through the skin is challenging due to the presence of the stratum corneum, the main barrier of substances. For this reason, the goal of this research was the combination of omega-3 (*ω*-3) and a dextran sulfate assembly in a nanostructure form, which allows passage through the skin and improves the bioavailability and the therapeutic profiles of active molecules, such as imiquimod. Here we report a new colloidal system, named dextran nanocapsules, with *ω*-3 in its nucleus and a coat made of dextran sulfate with a size ~150 nm, monomodal distribution, and negative zeta potential (~−33 mV). This nanosystem encapsulates imiquimod with high efficacy (~86%) and can release it in a controlled fashion following Korsmeyer–Peppas kinetics. This formulation is stable under storage and physiological conditions. Furthermore, a freeze-dried product could be produced with different cryoprotectants and presents a good security profile in the HaCaT cell line. Ex vivo assays with newborn pig skin showed that dextran nanocapsules promote transdermal delivery and retention 10 times higher than non-encapsulated imiquimod. These promising results make this nanosystem an efficient vehicle for imiquimod transdermal delivery.

## 1. Introduction

Imiquimod is an imidazoquinoline heterocyclic amine that belongs to a class of drugs known as immune response modifiers [1,2]; it is available in the market via a cream called Aldara^®^ at 5% concentration for the treatment of genital and perianal warts, but recently this molecule has been studied for use on different skin conditions, such as psoriasis and actinic keratosis [3]. Imiquimod has an efficient response as an immunomodulator in the treatment of different types of cancer and is an adjuvant of immunological response [4,5,6]. 

Currently, nanotechnology is searching for a way to encapsulate this kind of active molecule in nanoscale colloidal systems with specific physicochemical characteristics (<500 nm) [7], capable of compensating high degradation, low half-life, and poor penetration through the physical barriers of the human body [8,9]. Specifically, in the case of imiquimod, its incorporation in a nanosystem could allow it to have a better solubility profile and increase its bioavailability compared with conventional pharmaceutical formulations, which would open the possibility of using a lower amount to obtain the same therapeutic effect [10]. Currently, polymeric nanocapsules (NC) of chitosan and polysorbate have been used for the transdermal delivery of imiquimod in vaccinations or in the treatment of cervical cancer [11,12,13]. 

Nanotechnology not only studies the incorporation of active molecules in nanosystems but also allows the possibility of using a different administration route than commonly used ones (oral and parenteral), such as the transdermal route. The transdermal route permits the delivery of molecules with minimal invasiveness on the skin’s surface and can reach system circulation through cutaneous portals or other mechanisms [14,15], which are highly accepted by patients. But getting past the skin is challenging due to the presence of the stratum corneum, a sublayer of proteins and lipids arranged in a way referred to as “brick and mortar”, which gives it low permeability against foreign substances [16,17]. To improve this transdermal passage, the use or development of different excipients with properties that facilitate the flow of drugs through this barrier has been studied [18], such as polymers, surfactants, and fatty acids such as omega 3 (*ω*-3). These are capable of reducing the resistance of the lipid bilayer of this organ, thus allowing drugs to penetrate the lipids of this barrier [19]. 

With these antecedents, we can say that there is a fundamental challenge in administering imiquimod through the transdermal route for use in a wide variety of treatments. To address this challenge, this study focused on two important characteristics for crossing the stratum corneum. First, we used materials with chemical penetration-enhancing capacities, such as *ω*-3. Second, we used those materials that are capable of being assembled in nanometric structures. For this reason, in the present study, dextran NC with components that improve transdermal penetration and modify the release of imiquimod were designed. Ex vivo assays showed the capacity of this novel nanosystem loaded with imiquimod to cross the stratum corneum. 

## 2. Materials and Methods

### 2.1. Materials 

Acetone, potassium chloride, ethanol, methanol, sodium acetate, acetic acid, and imiquimod were obtained from Merck (Darmstadt, Germany). Dextran sulfate sodium from *Leuconostoc* spp. (6.5–10 KDa), oleic acid, glucose D+, dehydrate trehalose D+, resazurin, and triton-X-100 were purchased from Sigma Aldrich (Darmstadt, Germany). The HaCaT cell line was obtained from Dr. Borzutzky S.; Associate Professor of Medicine (Pontificia Universidad Católica de Chile). RPMI 1640 was acquired from Biological Industries (Beit HaEmek, Israel). Lipoid S75^®^ was purchased from Lipoid (Ludwigshafen, Germany). Varisoft PATC^®^ and *ω*-3 fatty acid were kindly donated by Evonik (Essen, Germany) and Golden Omega SA (Arica, Chile), respectively.

### 2.2. Preparation of Nanosystem

Nanosystems were designed using the solvent-displacement method [20]. The first nanosystem elaborated was a nanoemulsion (NE), with an oil phase that consisted of *ω*-3, oleic acid, lipoid S75^®^, and Varisoft PATC^®^ dissolved in 750 μL of ethanol and 4.25 mL of acetone and an aqueous phase consisting of 10 mL of Milli-Q water. Once the oil phase was prepared, this was directly added to the aqueous phase and mixed by magnetic stirring for 10 min. Finally, the organic solvents were eliminated by evaporation (Rotavapor Heidolph, Germany) to a constant volume of 5 mL. For the preparation of the dextran nanocapsules (DX NC) in a two-step methodology, 2 mL of the isolated NE was incubated with 2 mL of dextran sulfate solution (2 mg/mL) for 30 min. 

**Figure 1:**
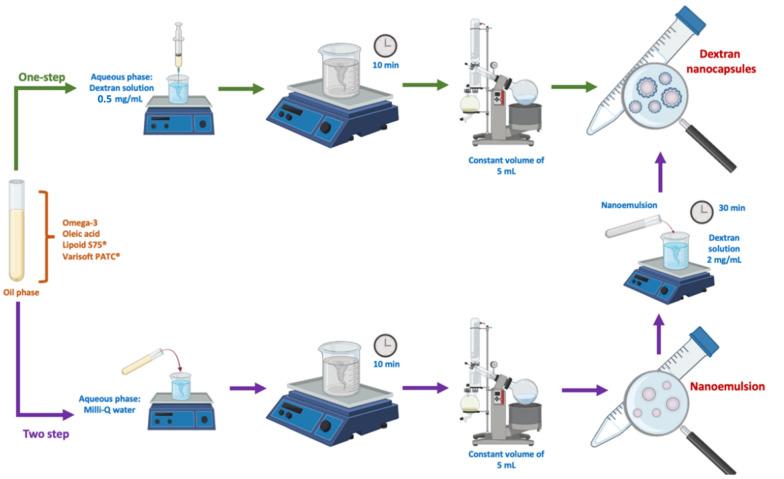
Process of the elaboration of the nanosystem. Created with BioRender.com (accessed on October 2022).

For the elaboration of the DX NC in one step, the same organic phase described previously for NE was used, but the aqueous phase employed was a solution of dextran sulfate in a concentration of 0.5 mg/mL. The organic phase was added directly or by pouring over the aqueous phase (pressure) using a disposable syringe (DX NC*). The organic solvents were eliminated by evaporation (Rotavapor Heidolph, Germany) to a constant volume of 5 mL (Figure 1).

Nanosystems were isolated by centrifugation (Universal 320 R, Hettich). To do so, 2 mL of the sample was deposited in Amicon^®^ (10,000 MCWO) centrifugation tubes. The conditions used were 4000 RPM for 30 min at 4 °C. Once the process finished, the supernatant was resuspended in an equal volume of Milli-Q water.

### 2.3. Physicochemical Characterization of the Nanosystem 

The hydrodynamic diameter, polydispersity index, and zeta potential of the nanosystem were determined by Dynamic Light Scattering (DLS) and laser doppler anemometry (Zetasizer^®^, NanoZs, Malvern Instruments, Malvern, UK) respectively. For the measure of size and polydispersity index, the samples were diluted in Milli-Q water, while for the measure of zeta potential, the samples were diluted in KCl (1 mM). 

Scanning Transmission Electron Microscopy (STEM) was used to study the morphology of the nanosystems. The samples were added to a carbon-coated copper grid and tinted with 2% phosphotungstic acid (*w*/*w*) solution.

### 2.4. Encapsulation of Imiquimod 

To incorporate imiquimod into the dextran NC (DX NC-imq*), a solution of imiquimod in oleic acid at 48 mg/mL was prepared which replaced the volume used by oleic acid in the formulation (Section 2.2). Then the same preparation method that incorporated the organic phase by pressure as described above (Section 2.2) was followed and their physicochemical characterization was studied (Section 2.3). The imiquimod concentration in the final formulation was 0.192 mg/mL. 

The efficiency of encapsulation of the imiquimod in the nanosystem was quantified using high-performance liquid chromatography with a mass detector (HPLC-MS/MS, Ekspert TM ultraLC 100, Danaher Corporation, AB Sciex, SA, Washington, DC, USA). A Supelco Ascentis^®^ 5 um Silica Column (2.1 mm × 100 mm) and stepped gradient method were used for the analysis. Methanol and ammonium acetate 1:9 constituted the mobile phase, with a flow of 0.8 mL/min. The column was thermoregulated at 40 °C and the samples at 15 °C. The injection volume was 10 μL. With the conditions described above, the time of retention of imiquimod was 2 min, and the total time of each chromatographic run was 4 min. 

A calibration curve was used each time the samples were analyzed to quantify imiquimod. The concentrations of imiquimod used were 10, 20, 30, 40, 50, 60, 70, and 80 PPB, using methanol as solvent. 

Imiquimod encapsulation in the NC was determined by calculating the ratio between the amount of imiquimod in the isolated NC (DX NC-imq*-I) and the amount of imiquimod in the non-isolated NC (DX NC-imq*).

### 2.5. Imiquimod Release

For this assay, horizontal diffusion cells (0.78 cm^2^ area, PermeGear, Inc., Hellertown, PA, USA) were separated by cellulose dialysis membranes (43 mm high, Sigma-Aldrich, Darmstadt, Germany). The donor cells were filled with 0.175 mL of the isolated nanosystem with imiquimod in their core (DX NC-imq*-I) and 3.325 mL of acetate buffer pH 5.5, ensuring sink conditions. The receptor solution was 3.5 mL of acetate buffer with a pH of 5.5. The system was maintained at 37 °C and protected from light. 

The samples were obtained at different times (1, 3, 5, 7, 10, 22, and 24 h) in a volume of 2 mL. Each time the volume was replenished to maintain sink conditions throughout the assay. Finally, the samples were diluted with methanol to quantify imiquimod by HPLC-MS/MS (Section 2.4) [21]. A solver program was used for kinetic release profile analysis [22]. 

### 2.6. Stability Assays of Dextran Nanocapsules 

For the study of the stability of DX NC* under physiological conditions (PBS pH 7.2, 37 °C), their physicochemical characterization (size and polydispersity index) was evaluated at different times (0, 0.25, 0.5, 1, 1.5, 2, 4, 6, 8, and 24 h). 

At storage conditions (4 °C), the stability of DX NC* and DX NC-imq* was analyzed by the measure of their physicochemical characterization (size, polydispersity index, and zeta potential) at different times (0, 1, 2, 3, 4, 8, 12, and 16 weeks). 

### 2.7. Freeze-Drying Studies 

To elaborate the freeze-dried product of the nanosystem, the need to use a cryoprotectant was studied. For this purpose, DX NC-imq* were mixed with different volumes of Milli-Q water or were diluted with different cryoprotectant solutions at the same volume with varied concentrations. Glucose and trehalose at 5% and 10% (*w*/*w*) were chosen for the assay. A mixture of 1 mL of the samples was frozen at −80 °C for two hours and then placed in a freeze dryer (FreeZone^®^, Labconco, Kansas City, MO, USA). The process conditions were −50 °C and 0.1 mbar of pressure for 24 h. Once the process of freeze-drying was over, the samples were stored in a desiccator at room temperature at 4 °C. All the samples were protected from light. 

To study the stability of the freeze-dried DX NC-imq*, the samples were resuspended in 1 mL of Milli-Q water at different times (0, 2, 4, 8, and 12 weeks) to measure their physicochemical characterization (size, polydispersity index, and zeta potential) according to Section 2.3. 

### 2.8. Studies of Cellular Viability 

To evaluate the cell viability of the designed DX NC* and DX NC-imq*, HaCAT cells were cultured in RPMI 1640 supplemented with 5% *w*/*w* fetal bovine serum (FBS) and 5 mL of penicillin (10,000 U/mL)/ Streptomycin (10 mg/mL) at 37 °C in a humidified atmosphere containing 5% carbon dioxide. 

For the assays, 100,000 cells were seeded per well in a 96 well-plate and incubated for 24 h. Different concentrations (30.4–0.23 mg/mL) of DX NC were diluted in RPMI 1640 and incubated with the cells for 4 h. Triton-X-100 (1:10 dilution in RPMI 1640) was used as a positive control and RPMI 1640 as a negative control. After this time, the medium with the treatment was removed, and the cells were incubated for 24 h with fresh RPMI 1640. Once the incubation finished, 20 μL of resazurin was added to each well and incubated for 4 h. The absorbance was quantified at 570 and 600 nm (Plate reader, BioTeK synergy HT, Waltham, MA, USA). The cell viability was calculated using the following Equation (1):

(1) $$\%\;\mathrm{cell}\;\mathrm{viability}=\frac{\left(\varepsilon 0X\right)\lambda 2A\lambda 1-\left(\varepsilon 0X\right)\lambda 1A\lambda 2\;\mathrm{samples}}{\left(\varepsilon 0X\right)\lambda 2A{}^{\circ}\lambda 1-\left(\varepsilon 0X\right)\lambda 1A{}^{\circ}\lambda 2\;\mathrm{negative}\;\mathrm{control}}\times 100\;$$

λ1 = 570 nm; λ2 = 600 nm
(ε0X)λ2 = Molar Extinction coefficient for resazurin at 600 nm = 117.216.
(ε0X)λ1 = Molar Extinction coefficient for resazurin at 570 nm = 80.586.
Aλ1 and Aλ2 = Observed absorbance of samples.
A°λ1 and A°λ2 = Observed absorbance of negative control.

### 2.9. Studies of Transdermal Penetration of Imiquimod

The absorption ability of DX NC-imq* in the skin was studied through ex vivo experiments using different portions of newborn pig skin [21,23], since they are similar to human skin in lipophilicity, thickness and histological structure [24]. The skin was extracted and stored at −20 °C before use. The samples of skin were cut into small pieces and placed into the vertical Franz cells (area 4.15 cm^2^, Laboratory Glass Apparatus Inc., Berkeley, CA, USA), where the epidermis was in contact with the donor compartment. The system for the study was set up with six Franz cells connected by a water bath thermoregulated at 37 °C and with constant agitation (300 RPM). 

The donor solution was 1 mL of DX NC-imq* or a solution of imiquimod in acetate buffer at a pH of 5.5 as the control. The receptor solution was an acetate buffer with a pH of 5.5. The samples were obtained by removing the total volume of the receptor cells at 7 and 24 h and the volume was replenished with the acetate buffer, maintaining the constant agitation and 37 °C of temperature. Once the samples were collected, they were put in an oven (Fisher Isotemp^®^ Oven Senior Model, Germany) at 50 °C in order to evaporate the acetate buffer. Finally, the evaporated samples were reconstituted in methanol to be quantified by HPLC-MS/MS as described in Section 2.4.

When the absorption studies were finished, the skin was removed from the Franz cells, carefully washed with methanol, and immersed in 15 mL of methanol for 24 h. After that, the skin was discarded, and the methanol samples were concentrated by total evaporation in an oven and reconstituted with methanol to be quantified the same way as recently described.

### 2.10. Statistical Analysis 

The statistical method selected to compare the results of transdermal assays was the t-student test with a confidence interval of 99%. 

## 3. Results and Discussion 

### 3.1. Design and Characterization of the Nanosystems

This study aimed to design a novel nanosystem to deliver imiquimod by the transdermal route. To do so, it used a simple and rapid elaboration method that does not use excessive energy, simultaneously allowing it to achieve a reproducible system with nanometric size, known as solvent displacement [25]. 

The excipients used in the nanosystem design were selected because of their widespread use in the pharmaceutical and cosmetic industries and their suitable profile of security and toxicity. Examples of this use include lecithin, which has been used for parenteral nutrition, and Varisoft PATC^®^ which has been implemented as a surfactant in shampoos and conditioners [26,27]. The oil phase of the nanosystem is composed of *ω*-3, an innovative excipient that has been studied in the field of nanotechnology for its capacity to act as a chemical enhancer of transdermal penetration. Furthermore, its solubilizer, anti-inflammatory, and moisturizer properties make it even more attractive for its administration on the skin [28,29]. The oil phase is also composed of oleic acid, a long-chain monounsaturated fatty acid sourced from different vegetal and animal sources, which similarly to *ω*-3, acts as a chemical enhancer of transdermal penetration [30,31]. Specifically, for the design of the NC, it was decided to use dextran sulfate as a coat because this biopolymer has important qualities for synthesizing nanomaterials, such as excellent solubility, biocompatibility, and biodegradability [32,33]. This polymer has been recently used in the design of a nanosystem for the oral route [34] and it has been studied in the transdermal delivery in the formation of a hydrogel for the delivery of dexamethasone [34]. 

The first nanosystem designed was a NE (Table 1) with a nanometric size (<200 nm) and a polydispersity index (PI) <0.3, which indicated a single monomodal distribution of particle size [35]. The zeta potential of the NE was positive, associated with the presence of the quaternary amine group in the structure of Varisoft PATC^®^ [26]. 

This positive charge allowed covering this NE with a polymer that has a negative charge. For this procedure, it was necessary to isolate the NE, and this process did not produce a change in the physicochemical characterization of the NE (NE-I; Table 1). DX-NC (two-Step) was produced by incubating dextran sulfate with primary NE-I for 30 min, and its physicochemical characterization showed an increase in size (~200 nm), while maintaining its monomodal distribution (IP < 0.3) and as seen in other formulations, the polymer is capable of changing zeta potential, crossing through a positive value to a negative value [36,37], which is associated with the presence of the sulfate group in the structure of dextran sulfate that covers the NE-I, (Table 1) [38,39]. 

Currently, industries are more interested in a manufacturing process having a continuous flow that operates under steady conditions and allows preparation of well-mixed phases [38]. For this reason, DX-NC was prepared in one step, in which the oil phase was directly poured over a dextran sulfate solution. The new NC showed a nanometric size, one population of this parameter, and a negative zeta potential, associated with the presence of dextran sulfate that was capable of adhering to a surface and changing this parameter to a negative value (Table 1). This work continued in the design since NC DX presents a greater size than DX-NC (two-step), and likewise, it has been shown that intercellular spaces of the lipid bilayer in the skin have a dimension close to 100 nm [39]; therefore, to obtain a similar size, nanosystems were prepared applying high manual pressure (DX NC*).

The use of pressure in the formulation allowed it to achieve DX NC with a size of ~181 nm, with a monomodal size and negative zeta potential (Table 1). This decrease in size in comparison with other NC can be associated with the pressure that multiple nanodrops was able to generate with almost no growth of them by incorporating the oil phase quickly and turbulently over the aqueous phase [40,41]. The other experiments continue with the DX NC* because this nanosystem had the smallest size, and its value (~181 nm) is close to the intercellular spaces of the skin, making it more promising for the transdermal delivery of imiquimod. 

The incorporation of imiquimod in the formulation was achieved by previously solubilizing the active molecule in the oleic acid that makes up part of the oil phase of the NC (Section 2.2). As seen in Table 1, the presence of imiquimod in the DX NC-imq* did not affect the physicochemical characterization, maintaining a similar value to DX NC*. Additionally, the isolated method did not modify the characterization (DX NC-imq*-I) as the formulations described before (Table 1). The encapsulation efficiency of imiquimod in the nanosystem was around 86 ± 7%, which indicated that the mix of oleic acid and *ω*-3 used is an excellent excipient for solubilized imiquimod, and was incorporated in the oil phase successfully [21,42]. Finally, this encapsulation percentage of imiquimod in DX NC* was better than other reported NC prepared using the same method with other polymers as coats and similar excipients [21,43]. 

**Figure 2:**
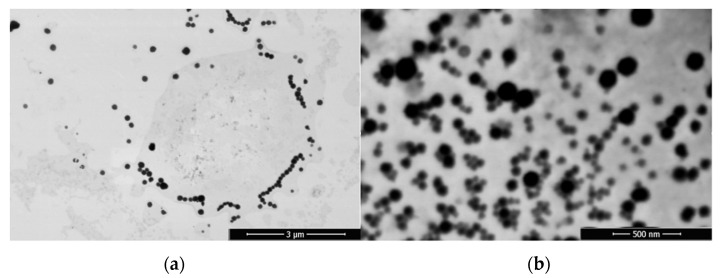
Images of (**a**) nanoemulsion and (**b**) dextran nanocapsules with imiquimod in their core (DX NC-imq*) obtained by scanning electron microscopy (STEM). * Formulation elaborated under pressure.

The morphology of the nanosystems was analyzed by STEM, as shown in Figure 2. DX NC-imq* presents a spheric form of nanometric and monomodal size, confirming the information obtained by DLS analysis [44]. 

### 3.2. Imiquimod Release Profile 

**Figure 3:**
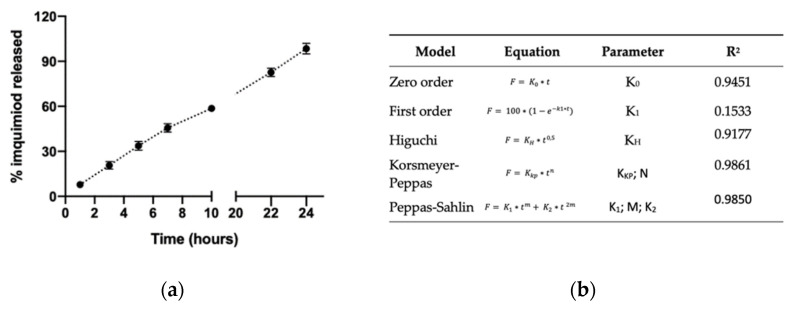
(**a**) Profile of imiquimod released from dextran nanocapsules with imiquimod in their core in acetate buffer pH = 5.5 at 37 °C for 24 h (**b**) Release model studies for the delivery of imiquimod from the dextran nanocapsules (Mean ± SD, n = 3).

The release profile of the imiquimod encapsulated in the nanosystem was studied as described in Section 2.5 at 37 °C. This temperature was used in the assay because we expect that the formulation crosses the skin and then releases the imiquimod once it is in the dermis. In this way, the imiquimod reaches systemic circulation. The skin is the organ responsible for maintaining a corporal temperature close to 37 °C [45,46]. Horizontal Franz cells were used, with a superficial contact area of 0.78 cm^2^, because these facilitate the exact extraction of the volume of the sample and have been used in other release assays [21,47]. Figure 3 shows imiquimod released from the core of DX NC and as can be seen in the first hour, less than 10% of the encapsulated imiquimod was released, but as time passed, this percentage increased, and in the seventh hour, more than 50% was released; once the experiment was over, almost 100% of imiquimod was released from the nanosystems. This shows that the amount of imiquimod released was greater than the chitosan NC designed by other researchers, which only released 40% of encapsulated imiquimod [21]. This indicates that DX NC-imq* allows the controlled release of imiquimod and prolongates the effect of the active molecule in comparison with other nanosystems that present a burst effect in the first hours under study [48]. The burst effect can be described as the immediate release of a high amount of the active molecule once the formulation is placed in the release medium [49]. This did not happen with the designed nanocapsule, which instead produced a slow delivery of imiquimod. This effect can be attributed to the presence of different mechanisms, such as erosion and diffusion. The former can be described as the degradation of the polymer due to the pH of the medium, the content of the polymer, and water absorption. Erosion is regulated by the type of polymer, internal bonding, shape, and size of the nanosystem [50]. On other hand, the diffusion mechanism depends on the concentration gradient where the molecules pass through the polymer, which acts as a barrier and restricts the movement of the drug. The drug release rate depends on the physical and chemical characteristics of the membrane, the amount of the drug-loaded and the membrane (polymer) thickness [50,51]. However, we cannot say which of the mechanisms is responsible or predominant for the controlled delivery, but the dextran sulfate used as corona effectively coated the oil nucleus (*ω*-3) and allowed it to keep this active molecule inside the nanosystem for more time. 

This assay permitted studying which of the drug release models could adjust to the release profile of imiquimod in these nanosystems; the models studied were: zero-order kinetic, first order-kinetic, Higuchi, Korsmeyer–Peppas and Peppas–Sahlin of drug release. The best fit was Korsmeyer–Peppas with *R*^2^ = 0.9861 and *n* = 0.65, which indicates that the release of imiquimod follows a non-fickian or anomalous transport governed by diffusion [52]. This fashion following Korsmeyer–Peppas has been fitted to other nanosystems (NC) that release cloxacillin-benzathine [53].

### 3.3. Stability of the Dextran Nanocapsules 

**Figure 4:**
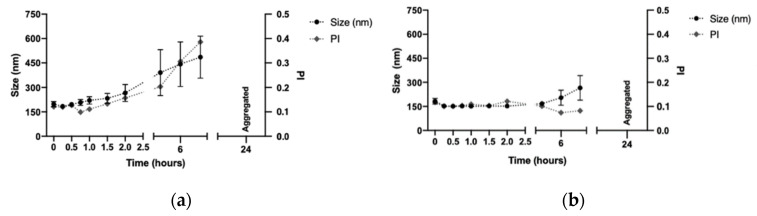
Stability of dextran nanocapsules (DX NC*) with or without imiquimod in their core. Conditions: PBS (pH 7.2), 37 °C. (**a**) Size and polydispersity index of DX NC*. (**b**) Size and polydispersity index of DX NC-imq*. Imq: imiquimod. IP: polydispersity index. * Formulation elaborated applying high manual pressure. (Mean ± SD, n = 3).

The respective stabilities of DX NC* and DX NC-imq* were studied by evaluating their physicochemical characterization by submitting them under physiological and storage conditions (Section 2.5). As shown in Figure 4, both nanosystems were stable under physiological conditions for at least 8 h; the change in their size and polydispersity index after 8 h could be associated with the stress of submitting the NC during this assay. The saline medium and the work temperature (37 °C) could produce a phenomenon known as Ostwald ripening, in which smaller particles, due to their better solubility, become absorbed over greater particles, and reflect bigger sizes that are not in the same range [54]. Additionally, at the same time, the temperature could increase the possibility of NC suffering collisions with each other and to the aggregate [55]. 

**Figure 5:**
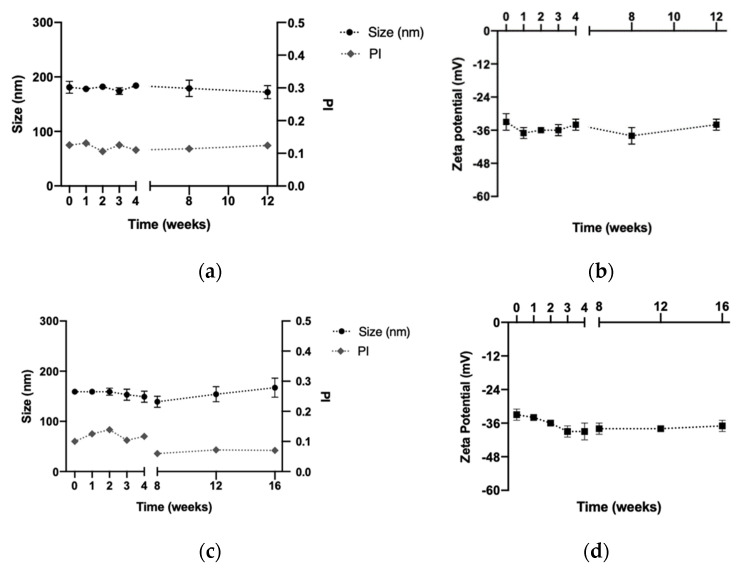
Stability of the dextran nanocapsules (DX NC*) with or without imiquimod in their core at 4 °C. Conditions: 4 °C. (**a**) Size and polydispersity index of DX NC*. (**b**) Zeta potential of DX NC*. (**c**) Size and polydispersity index of DX NC-imq*. (**d**) Zeta potential of DX NC-imq*. Imq: imiquimod. PI: polydispersity index. * Formulation elaborated applying high manual pressure. (Mean ± SD, n = 3).

DX NC* and DX NC-imq* were stable in storage conditions. Specifically, DX NC* remained stable for 3 months (Figure 5A,B) and DX NC-imq* did for 4 months (Figure 5C,D). The maintenance of their physicochemical properties can be attributed to the electrostatic repulsion generated by the sulfate group in the DX polymer, which acts as a coat [56,57]. Furthermore, the possibility of developing a freeze-dried product of the DX NC-imq* was studied in order to enhance the stability of this type of nanosystem at room temperature or at 4 °C [58].

### 3.4. Freeze-Drying Studies 

**Figure 6:**
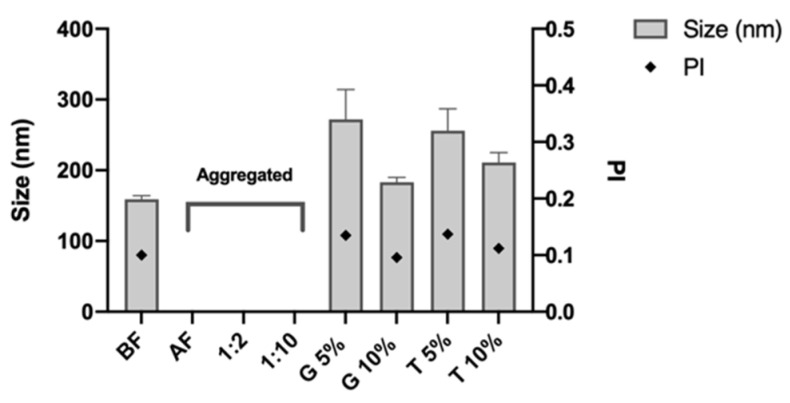
Physicochemical properties (size and polydispersity index) of dextran nanocapsules with imiquimod in their core (DX NC-imq*) once were subjected to freeze-dry process. BF: before freeze-drying. AF: after freeze-drying. 1:2 and 1:10: dilution in Milli-Q water. G: Glucose. T: Trehalose. PI: polydispersity index. * Formulation elaborated applying high manual pressure. (Mean ± SD, n = 3).

To produce a freeze-dried product, DX NC-imq* were diluted with different proportions of Milli-Q water or cryoprotectants and then freeze-dried for 24 h (Section 2.6). As can be seen in Figure 6, DX NC-imq* freeze-dried and diluted at different concentrations of Milli-Q were aggregated, which makes nanosystems incapable of maintaining their physicochemical characterization, probably due to the stress to which the samples were subjected. This occurred especially in the process of freezing the nanosystems, where the formation of water crystals gives the result of a saturated solution of the remaining components, which increases the possibility of aggregation [59]. This result indicated that cryoprotectants are necessary to keep the size and polydispersity index of DX NC [60].

Two sugars were studied in choosing the cryoprotectant. As shown in Figure 6, using glucose and trehalose could keep a nanometric size and PI of DX NC-imq because these excipients can form a matrix around the formulation, thus reducing molecular mobility and preventing interaction between the NC and the possibility of aggregation [61,62]. With these assays, we were able to observe that the conservation of the physicochemical properties of nanosystems could be dependent on the concentration of the cryoprotectant. For instance, the use of glucose and trehalose at 10% (Figure 6) managed to get a smaller size similar to the size of NC before freeze-drying. This phenomenon could endorse the theory that a specific cryoprotectant concentration can generate an efficient matrix around the nanosystems [63]. The slight difference in size we obtained with the cryoprotectants could be related to the different affinities for the formulation that these sugars possess, where glucose can protect nanosystems stabilized by polymers, and where trehalose has more affinity with nanosystems stabilized only with surfactants [64]. 

**Figure 7:**
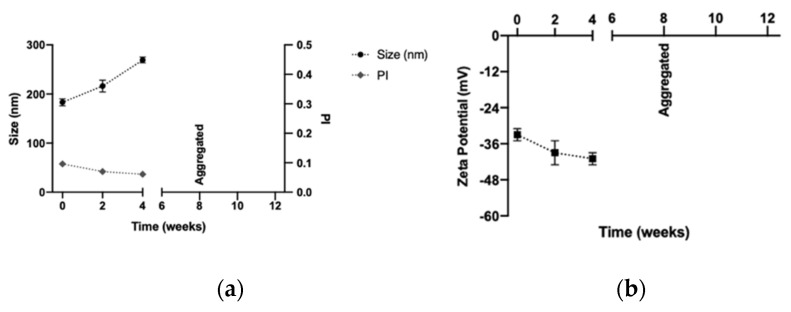

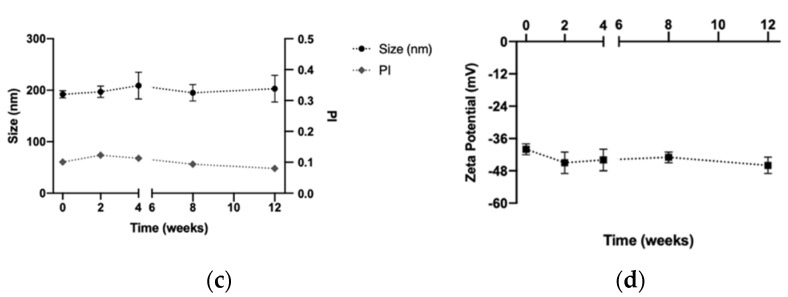
Stability of the dextran nanocapsules with imiquimod in their core (DX NC-imq*) subjected to freeze-drying and stored at room temperature. (**a**,**b**) Size, polydispersity index and zeta potential of DX NC-imq* freeze-dried with glucose at 10%, respectively. (**c**,**d**) Size, polydispersity index and zeta potential of DX NC-imq* freeze-dried with trehalose at 10%, respectively. PI: polydispersity index. * Formulation elaborated applying high manual pressure. (Mean ± SD, n = 3).

It was decided to continue the stability test with both sugars at a concentration of 10% at room temperature, as shown in Figure 7. The use of glucose could maintain the size, polydispersity index, and zeta potential for at least one month in storage at room temperature, but in the second month, the formulation was aggregated. This did not happen with the freeze-dried formulation with trehalose, which maintained its physicochemical proprieties for at least three months under these conditions. 

**Figure 8:**
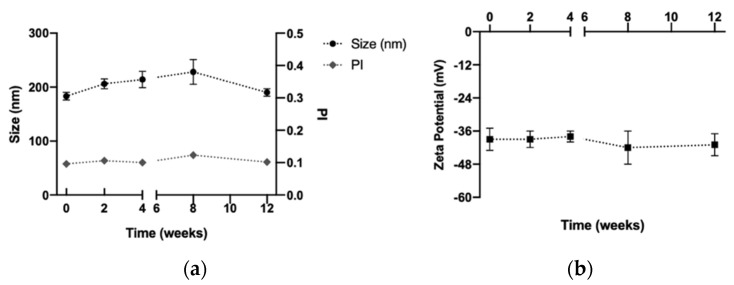
Stability of the dextran nanocapsules with imiquimod in their core (DX NC-imq*) subjected to freeze-dry with glucose at 10% and stored at 4 °C. (**a**,**b**) Size, polydispersity index and zeta potential of DX NC-imq* freeze-dried with glucose at 10%, respectively. PI: polydispersity index. * Formulation elaborated applying high manual pressure. (Mean ± SD, n = 3).

The aggregation of the DX NC-imq* which used glucose as a cryoprotectant could be associated with the loss of the amorphous form of glucose since its structure can change to a crystalline form at room temperature under the storage conditions to which they were submitted [65]. Similar nanosystems freeze-dried with glucose as a cryoprotectant have been shown through studies to lose their stability over time during storage at room temperature [21]. However, trehalose has low chemical reactivity and can keep its amorphous structure during storage at room temperature [60]. In the last stability assay, glucose’s capacity to maintain the physicochemical characterization of DX NC-imq* in storage conditions at 4 °C was studied. As shown in Figure 8, DX NC-imq* was stable for at least three months when we changed the storage conditions, because the crystallization of the glucose was avoided. Therefore, DX NC-imq* demonstrated good stability during this time, and can be freeze-dried while maintaining its physicochemical characteristics for at least 3 months. Similar nanosystems have been capable of maintaining their physicochemical characteristic in time when undergoing the freeze-drying process, as has happened with DX NC*-imq [66], and we expect that the designed nanosystem will remain stable in time.

### 3.5. Cellular Viability 

**Figure 9:**
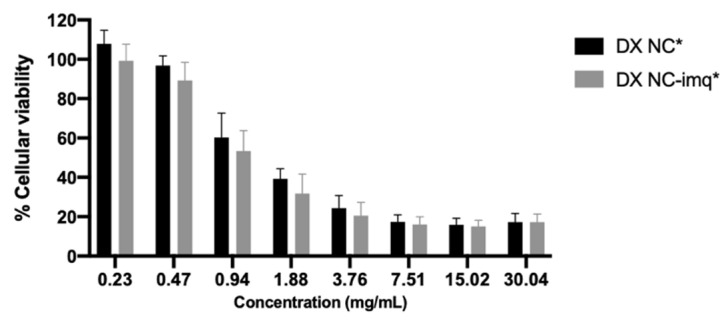
Viability of HaCat cell line after 4 h of exposure at different concentrations of dextran nanocapsules (DX NC*). DX NC-imq*: dextran nanocapsules with imiquimod in their core. * Formulation elaborated applying high manual pressure. (Mean ± SD, n = 3).

This assay was performed in order to study the effect of DX NC* and DX NC-imq* on the HaCat cell line. These kinds of cells are spontaneously transformed human keratinocytes and this test was performed because it gives us an idea of the biocompatibility of the nanosystems with human skin [67]. Figure 9 shows that there are concentrations in both nanosystems that do not affect the growth of HaCat cells. However, at a high concentration of both NC, we can see inhibition of the cell growth.

The inhibition of the growth of HaCat cells can be attributed to the physiochemical proprieties of the nanosystems. As mentioned in Section 3.1, these NC present a nanometric size, and it has been shown that smaller size provides a greater surface area, which increases the possibility of reactive groups interacting with cells [68]. Likewise, the excipients used in the NC can contribute to cell growth inhibition, as with Varisoft PATC^®^. This surfactant is a cationic molecule with a positive charge capable of interacting with the phospholipids in the cellular membranes, causing difficulty in maintaining cell structure, physiological activity, and proliferation [69].

It is important to mention that these assays give us an in vivo correlation of what could happen with the administration of DX NC, and in this case, the nanosystems were incubated with a limited number of cells, much less than the number of cells that formulations would be exposed to in the skin in future administration [70]. Likewise, the concentration used in a future administration will be similar to the ones used for the well that presented a higher viability, which allows us to describe both NC with a good safety profile.

### 3.6. Transdermal Penetration of Imiquimod

**Figure 10:**
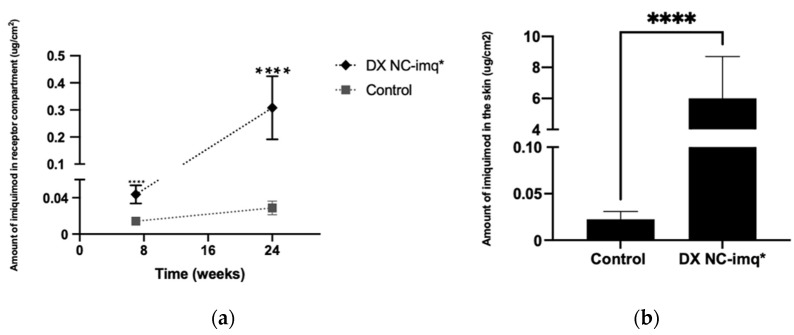
Studies of transdermal permeation and retention of imiquimod in the designed nanosystem. (**a**) Transdermal penetration of imiquimod carried in DX NC-imq*. (**b**) Imiquimod skin retention at the end of the transdermal studies. DX NC-imq*: dextran nanocapsules with imiquimod in their core. * Formulation elaborated applying high manual pressure. (Mean ± SD, n = 6). **** Statistically significant (*p* < 0.01).

The main challenge we faced in this research was determining if Dx NC-imq* allows the transdermal penetration of imiquimod. To evaluate this, a system with six vertical Franz cells was mounted, which has a much larger contact area (0.78 v/s 4.25 cm^2^) than the horizontal cells used for the liberation studies. This larger area permits quantifying with greater certainty the amount of imiquimod that crosses the skin [71]. As shown in Figure 10A, a small fraction of free active molecule is capable of going through the skin due to its low molecular weight and high lipophilicity [42]. However, once imiquimod is encapsulated in DX NC-imq*, the amount of imiquimod able to cross the skin is statistically higher (*p* < 0.05), exceeding by ten times the amount of non-encapsulated drug at 24 h. This result can be associated with the presence of novel chemical penetration enhancers present in the nucleus of the nanosystem such as *ω*-3, which can disrupt the special arrangement of the lipids present in the skin [28,72]. The combination of this oily nucleus with sulfate dextran polymer allows the development of a vehicle with properties that make it capable of improving the passage of the active molecule through the physiological barrier (skin) [73]. Additionally, DX NC-imq* has a nanometric size very close to the size of the intercellular spaces present in the stratum corneum. This small size increases the probability of the nanosystem going through these spaces, crossing the lipidic bilayer and reaching the dermis [39]. It is worth mentioning that the NC designed has a negative potential zeta that can generate electrostatic repulsion with carboxylic residues of lipid and proteins present in the skin; in this way, some authors postulate that this physicochemical characteristic could promote its passage through the barrier [74,75]. For this reason, we can associate this result with the combination of excipients capable of being assembled in a nanocapsule with specific physicochemical characteristics that produce a high retention and passage of imiquimod through the skin.

The transdermal retention studied shows the amount of imiquimod encapsulated in the nanosystem that stays in the skin is 10 times greater than the amount of imiquimod in the control (Figure 10B). The imiquimod retained in the skin can act as a reservoir, with delivery from the nanocapsule across the skin and reaching the dermis or acting directly on the organ. This phenomenon can be attributed to the imiquimod being solubilized and encapsulated in a lyophilic nucleus coated by dextran sulfate, which makes it capable of changing its partition coefficient and improving its affinity for the skin [28]. 

These experiments showed that DX NC-imq* acts as a promising nanosystem for transdermal delivery since these NC achieve better penetration of imiquimod through the skin than nanosystems designed with similar excipients [2].

## 4. Conclusions

In the present work, it was possible to design a novel nanosystem (dextran nanocapsules with imiquimod in their core) with a size close to 159 nm, IP < 0.3, and a zeta potential close to −30 mV. These nanocapsules were designed with novel excipients that promote the transdermal penetration of imiquimod through the skin, such as omega-3 (*ω*-3) and dextran. Dextran nanocapsules presented an excellent capacity to encapsulate the active molecule (~86%) and showed controlled release through time following a Korsmeyer–Peppas kinetic. It is important to mention that this nanosystem has an excellent security profile proven in viability assays with the HaCat cell line and is stable for 8 h in phycological condition and 4 months at storage conditions. Additionally, it is possible to generate a freeze-dried product with different cryoprotectants of the nanosystem that maintain their phytochemical characterization in time. Finally, ex vivo studies showed this new formulation promote transdermal delivery and retention 10 times higher than non-encapsulated active molecule. With these results, we can say that dextran nanocapsules are an efficient vehicle for transdermal delivery of imiquimod. 

## Figures and Tables

**Table 1:** Physicochemical characterization (mean particle size, polydispersity index (PI), and zeta potential (ZP)) of the nanosystems designed using either a one-step or two-step methodology. NE: nanoemulsion. DX NC: dextran nanocapsules. I: Isolated nanosystems. * Formulation elaborated applying high manual pressure. Imq: imiquimod (Mean ± SD, n = 3).

Formulation	Size (nm)	PI	ZP (MV)
NE	179 ± 5	0.046	+47 ± 4
NE-I	175 ± 5	0.118	+37 ± 4
DX NC (two-step)	202 ± 8	0.077	−54 ± 2
DX NC (two-step)-I	200 ± 2	0.084	−54 ± 1
DX NC	264 ± 13	0.094	−38 ± 2
DX NC*	181 ± 11	0.125	−37 ± 3
DX NC-imq*	159 ± 5	0.100	−37 ± 2
DX NC-imq*-I	148 ± 8	0.100	−37 ± 2
